# Adult-onset Still’s Disease: A Case Report

**DOI:** 10.31729/jnma.4844

**Published:** 2020-02-29

**Authors:** Ashok Sapkota, Nirdesh Pokhrel, Jayaram Adhikari, Bishal Shrestha, Yoveen Kumar Yadav

**Affiliations:** 1 Department of Medical Oncology, B.P. Koirala Memorial Cancer Hospital, Bharatpur, Nepal; 2 Department of Internal Medicine, Bharatpur Samudayik Hospital, Bharatpur, Nepal; 3 Tuberculosis Care and Control Project, Nepal CRS Company, Kathmandu, Nepal

**Keywords:** *adult-onset stills disease*, *arthritis*, *ferritin*, *fever*

## Abstract

Adult-onset Still’s Disease is a rare auto inflammatory disorder of unknown etiology characterized mainly by high spiking fever, arthritis, evanescent rash and lymphadenopathy. It is a form of systemic onset juvenile rheumatoid arthritis that is encountered in adults, typically between 15-25 years and 36-45 years. We here describe a 28 years lady with fever, arthritis of multiple large joints, lymphadenopathy and rash, with negative Rheumatoid factor and evidence of past infection with Ebstein-Barr virus and Parvovirus B19. History, examination findings and investigations showed several features consistent with adult-onset Still’s disease along with high ferritin level. After exclusion of probable other diagnosis and use of Yamaguchi criteria, she was diagnosed with adult-onset Still’s disease. All the major and minor criteria of Yamaguchi for diagnosis were met. Her disease responded well with steroid, she achieved remission and is currently under maintenance therapy.

## INTRODUCTION

Adult-onset Still’s Disease (AOSD) is a chronic inflammatory disorder characterized by high fever, joint pain and nonpruritic rash. Still’s disease was initially described by George F. Still in 1896 as a form of chronic joint disease in children, resembling rheumatoid arthritis in adult.^1^ Later in 1971 Bywaters described illness starting in adult life resembling Still’s disease or sero-negative chronic polyarthritis of children.^2^ Its prevelance is about 0.16/100,000 cases, with bimodal age distribution, between 15-25 years and 36-45years.^3^ It is a diagnosis of exclusion. We here discuss a case of 28 years lady who is diagnosed as AOSD and currently under treatment.

## CASE REPORT

A 28 years lady presented to BPKMCH medical oncology OPD with complaints of fever, pain and swelling of multiple joints, recurrent sore throat and rash since 2 years. Fever was associated with chills and rigors, and was associated with nonpruritic rash in upper half of body. Lady complained of pain in left wrist joint, bilateral elbow and knee joint. Lady recalled history of sore throat 2 years back before the fever and joint pain started, however she denied any history of cough, shortness of breath, oral ulcers, photosensitivity or muscle weakness.

On physical examination, lady was pale, febrile, ill defined macular rash were present in upper half of body, left wrist joint was swollen and tender, multiple enlarged tender cervical, axillary and inguinal nodes along with hepatosplenomegaly was present.

Lady had visited multiple centres over period of 2 years and she was found to be started on steroids. She also agrees that joint pain and fever had temporarily subsided when she was on steroids. However due to lack of proper follow up in a particular centre and frequent use of homeopathic as well as over the counter drugs, her symptoms failed to respond to medication for a Relevant investigations were initiated (Table 1).

**Table 1:** Initial blood investigations and results.

Tests CBC	Results
Hemoglobin	6.7 g/dl
Total Leucocyte count	14,200/mm^3^
Neutrophils	93%
Lymphocytes	6%
Eosinophils	1%
Platelets	3,24,000/mm^3^
ESR	71 mm/hr
Peripheral blood smear	Microcytic hypochromic anemia, anisopokilocytosis Without any atypical cells or parasites
LFT	
Total Bilirubin	0.83 mg/dl
Direct Bilirubin	0.49 mg/dl
AST	33 U/L
ALT	24 U/L
ALP	492 U/L
LDH	854 U/L
Total protein	7.9 g/dl
Albumin	1.9 g/dl

CBC = complete blood count; ESR = erythrocyte sedimentation rate; LFT = liver function tests; AST = aspartate aminotransferase; ALT = alanine aminotransferase; ALP = alkaline phosphatase; LDH = lactate dehydrogenase.

**Figure 1:**
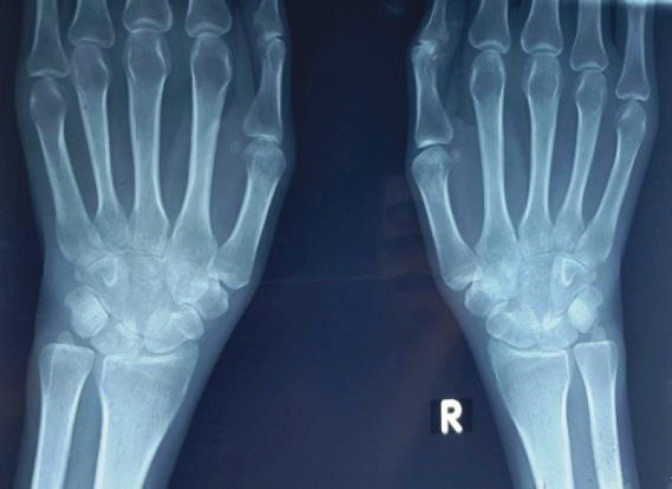
X-ray bilateral wrist joint showing reduced joint space in left.

Her serum iron profile was obtained to evaluate anemia, and a contrast enhanced computed tomography (CECT) of chest, abdomen and pelvis was done. X-ray of wrist joint was done which showed bone erosion with narrowed joint space (Figure 1).

**Figure 2a and 2b:**
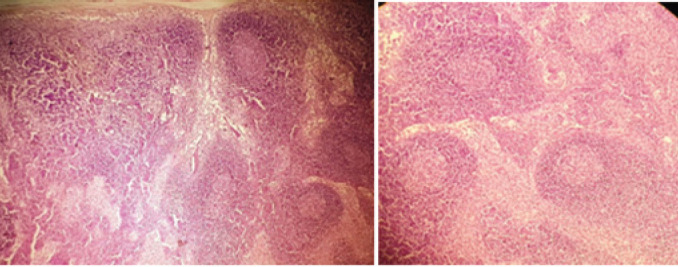
4 X and 10 X view of inguinal lymph node biopsy showing varying sized follicles with germinal center cells and sinus histiocytes suggestive of reactive lymphadenitis.

Her echocardiography was normal, serum ferritin level was significantly raised, CECT showed mild hepatosplenomegaly with multiple lymphadenopathies. In view of radiologically suspected lymphoma, lymph node biopsy and bone marrow aspiration (BMA) was done. BMA showed normocellular marrow with mild hyperplasia of eosinophils precursors without any atypical cells or parasites. Inguinal lymph node biopsy showed reactive lymphadenitis (Figure 2a and 2b).

The obtained investigations and clinical history, was found to meet all the major and minor Yamaguchi criteria for diagnosis of AOSD. Additional investigations were done to look for the presence of infections and rheumatic disorder (Table 2).

**Table 2:** Serum iron profile and viral serology.

Tests	Results
Serum iron profile	
Ferritin	> 16,500 ng/mL
Iron	83 μg/dL
Total Iron Binding	149 μg/dL
Capacity Transferrin saturation	55.7 %
Anti Nuclear Antibody, IFA ( HEP-2)	Minimal fuorescence
	Homogenous pattern
	Primary and end point titer 1:80
Viral serology	
Ebstein-Barr Virus	IgG: Positive, IgM: Equivocal
	Repeated IgM ( after 2 weeks ): Negative
Cytomegalovirus	IgG: Positive, IgM: Negative
Parvovirus B9	IgG: Positive, IgM: Negative
Rubella	IgG: Positive, IgM: Negative, High IgG Avidity
Monospot test for Heterophile antibody:	Negative

IFA = Indirect Immunofluoresecence assay; Ig = Immunoglobulin

Thus as per Yamaguchi criteria, after excluding possible malignancy, infection and rheumatic disease, patient was diagnosed with AOSD.

She was started on oral prednisolone at 1 mg/kg, blood transfusion was done and supportive care was given with analgesics and anti-pyretics. She was discharged with oral prednisolone 40 mg per day, weekly alendronate and Ibuprofen. At 1 month of follow up, patient was symptomatically better with, no episodes of fever or joint pain and rash. Prednisolone tapering was initiated, and she was started on 15 mg per weekly methotrexate. During follow up, patient was on 5 mg prednisolone per day, and there was no episode of fever or arthralgia. A repeat CBC at 2 months showed reduced ESR and leucocytosis. A repeat CECT showed significant reduction in number and size of lymphadenopathies. So the patient was continued on 5 mg prednisolone per day and 7.5 mg methotrexate per weekly. Patient is under regular follow up till date.

Patient is well counselled about possible complications of disease, risk of lymphoproliferative disease, in view of EBV positive lymphadenopathies and EBV reactivation that may occur with prolong immune suppressive drugs.

## DISCUSSION

AOSD is a rare auto inflammatory disorder of unknown etiology characterized mainly by high spiking fever, arthritis, evanescent rash and lymphadenopathy. AOSD is a diagnosis of exclusion. A high-sensitivity Yamaguchi classifcation criteria is useful in diagnosis of AOSD (Table 3).^4^

**Table 3:** Yamaguchi criteria for diagnosis of AOSD (sensitivity 96.2%, specificity 92.1%)

**Major criteria**	Fever 39 lasting >1 week Arthralgia or arthritis lasting > 2 weeks Typical non pruritic salmon-colored rash Leukocytes > 10,000/mm^3^ with granulocytes 80%
**Minor criteria**	Sore throat Lymphadenopathy Splenomegaly Abnormal liver function tests Negative testes for antinuclear antibody(ANA) and rheumatoid factor
**Exclusion criteria**	Infection Malignancy Other rheumatic disease
**Criteria for diagnosis of AOSD**	≥ 5 criteria with at least 2 major criteria and no exclusion criteria

Our patient’s symptoms, signs and her laboratory findings met all major and minor criteria for diagnosis of AOSD. Most common clinical features include: arthralgia ( 98-100%), fever >39 (83-100%), myalgia (84-90%), rash (87-90%), sore throat (50-92%).^5^ Arthralgia and arthritis is common finding in AOSD that may progress to destructive polyarthritis.^6^ Typical rash of AOSD is a evanescent, salmon coloured, maculopapular, usually nonpruritic predominantly involving proximal limbs and trunk.^7^ Nonsuppurative pharyngitis is a early feature in course of AOSD that can precede fever. Sore throat in AOSD is purposed to be due to underlying cricothyroid pericondritis.^8^ Lymphadenopathy is a common finding in AOSD observed in about 90% of patients and is a minor criteria in Yamaguchi criteria.^9^ Lymphadenopathy along with fever and leucocytosis can also be the presenting feature of lymphoma, so lymph node biopsy also has a important role in diagnosis of AOSD and to rule out probable malignancy. Anemia, leucocytosis with neutrophilia and reactive thrombocytosis is commonly seen in AOSD.^5,10^ It has been suggested that various infections may trigger disease in genetically susceptible host. In view of exclusion of probable infectious etiology as suggested by Yamaguchi criteria, viral serology obtained in this patient showed positive serology of EBV, CMV, parvovirus B19 and rubella. High IgG avidity pointed towards past rubella immunization. These viral infection might be the triggering factor in this particular patient. Laboratory findings like anemia, elevated ALP and LDH, reduced albumin, elevated ESR, CRP along with increased serum ferritin also suggest the possibility of AOSD. Serum ferritin more than 5 times the normal upper limit ( >1000 ng/ml) is a useful biomarker for AOSD.^11^

Non-steroidal anti-inflammatory agents (NSAIDS) are useful to control symptoms in early disease, or during diagnostic workup, corticosteroids are first line drugs and Disease modifying anti-rheumatic drugs (DMARDs) are useful when there is failure to achieve remission with corticosteroids, as steroid sparing agents or during maintenance therapy after remission.^6^

AOSD is a diagnosis of exclusion. It should be considered a differential in patients with long standing fever, musculoskeletal symptoms, lymphadenopathy with negative RA factor. The symptoms can be vauge due to use of over the counter drugs. A careful documentation of symptoms, exclusion of other differentials and use of Yamaguchi criteria can help in diagnosis. Serum ferritin level is a useful tool for diagnosis. A timely diagnosis and treatment along with proper counselling about regular follow up and disease status can improve the outcome of disease as well as quality of life of the patient.
